# Comparison of Common and Disease-Specific Post-translational Modifications of Pathological Tau Associated With a Wide Range of Tauopathies

**DOI:** 10.3389/fnins.2020.581936

**Published:** 2020-11-04

**Authors:** Fuyuki Kametani, Mari Yoshida, Tomoyasu Matsubara, Shigeo Murayama, Yuko Saito, Ito Kawakami, Mitsumoto Onaya, Hidetomo Tanaka, Akiyoshi Kakita, Andrew C. Robinson, David M. A. Mann, Masato Hasegawa

**Affiliations:** ^1^Department of Brain and Neuroscience, Tokyo Metropolitan Institute of Medical Science, Tokyo, Japan; ^2^Institute for Medical Science of Aging, Aichi Medical University, Aichi, Japan; ^3^Department of Neuropathology, Tokyo Metropolitan Institute of Gerontology, Tokyo, Japan; ^4^Department of Pathology and Laboratory Medicine, National Center Hospital, National Center of Neurology and Psychiatry, Tokyo, Japan; ^5^Department of Psychiatry, National Hospital Organization Shimofusa Psychiatric Medical Center, Chiba, Japan; ^6^Department of Pathology, Brain Research Institute, Niigata University, Niigata, Japan; ^7^Division of Neuroscience & Experimental Psychology, Faculty of Biology, Medicine and Health, School of Biological Sciences, The University of Manchester, Salford, United Kingdom

**Keywords:** tauopathy, tau, post-translational modifications, amyloid, fibril structure, cryo-EM, ubiquitination, phosphorylation

## Abstract

Tauopathies are the most common type of neurodegenerative proteinopathy, being characterized by cytoplasmic aggregates of hyperphosphorylated tau protein. The formation and morphologies of these tau inclusions, the distribution of the lesions and related metabolic changes in cytoplasm differ among different tauopathies. The aim of this study was to examine whether there are differences in the post-translational modifications (PTMs) in the pathological tau proteins. We analyzed sarkosyl-insoluble pathological tau proteins prepared from brains of patients with Alzheimer’s disease, Pick’s disease, progressive supranuclear palsy, corticobasal degeneration, globular glial tauopathy, and frontotemporal dementia and parkinsonisms linked to chromosome 17 with tau inclusions using liquid chromatography mass spectrometry. In pathological tau proteins associated with a wide range of tauopathies, 170 PTMs in total were identified including new PTMs. Among them, common PTMs were localized in the N- and C-terminal flanking regions of the microtubule binding repeats and PTMs, which were considered to be disease-specific, were found in microtubule binding repeats forming filament core. These suggested that the differences in PTMs reflected the differences in tau filament core structures in each disease.

## Introduction

Neurodegenerative tauopathies are characterized by cytoplasmic aggregates of hyperphosphorylated tau protein (Goedert et al., 1992; Buee et al., 2000; Lee et al., 2001). Among them, Alzheimer’s disease (AD), chronic traumatic encephalopathy (CTE), Pick’s disease (PiD), progressive supranuclear palsy (PSP), corticobasal degeneration (CBD), globular glial tauopathy (GGT), and argyrophilic grain disease (AGD) are sporadic tauopathies, while frontotemporal dementia and parkinsonisms linked to chromosome 17 with tau inclusions (FTDP-17T) is a familial disease caused by abnormalities in the tau gene, *MAPT* (Iwatsubo et al., 1994; Spillantini et al., 1997, 1998; Hutton et al., 1998; Poorkaj et al., 1998; Buee and Delacourte, 1999; Goedert and Hasegawa, 1999; Lee et al., 2001; Kovacs, 2015). Although the morphologies of tau inclusions and the distribution of the lesions differ, these diseases are neuropathologically defined by the characteristic tau pathologies observed in nerve cells and/or glial cells, and the formation of these tau aggregates and related metabolic changes in the cytoplasm are believed to be the cause of neurodegeneration (Goedert et al., 1992; Buee et al., 2000; Lee et al., 2001).

Expression of the *MAPT* gene is developmentally regulated by means of alternative splicing of the exons. In adult human brain, six tau isoforms with either three or four microtubule-binding repeats (3R or 4R) are expressed (Goedert et al., 1989). In AD brains, both 3R and 4R tau isoforms are accumulated in a hyperphosphorylated state as pathological inclusions called neurofibrillary tangles and neuropil threads, mainly in neuronal cells (Goedert, 1993; Goedert et al., 1996; Serrano-Pozo et al., 2011; Iqbal et al., 2016). Ultrastructurally, a unique twisted paired helical filaments (PHFs) or related straight filaments (SFs) with a diameter of 10–20 nm and periodicities periodicity of 80 nm are observed (Crowther and Wischik, 1985; Wischik et al., 1988a, b; Crowther et al., 1989; Goedert et al., 1989; Greenberg and Davies, 1990; Lee et al., 1991). On the other hand, in PiD, only 3R tau isoforms selectively accumulate in neuronal cells with SF morphology (15–18 nm), although some exceptions are have been reported (Falcon et al., 2018). In PSP, CBD, GGT, and AGD, 4R tau isoforms selectively accumulate in neuronal and glial cells, and the filaments in each disease have unique filamentous morphologies, such as SFs (13–14 nm) in PSP and wide, twisted filaments (20 nm) in CBD (Iwatsubo et al., 1994; Spillantini et al., 1997; Hutton et al., 1998; Poorkaj et al., 1998; Buee and Delacourte, 1999; Goedert and Hasegawa, 1999; Lee et al., 2001; Kovacs, 2015). Furthermore, it has been reported that CBD and PSP can be distinguished biochemically based on the characteristics of the C-terminal fragments (Arai et al., 2004) and the band patterns of protease-resistant fragments (Taniguchi-Watanabe et al., 2016), even though they are both 4R tauopathies.

The discovery of *MAPT* mutations in FTDP-17T established that abnormalities in tau expression and function can cause tau accumulation and neurodegeneration. The clinico-pathological phenotypes of the disease vary, but there is a strong correlation between the locations of the mutations and the neuropathology of the tau proteins (morphology of inclusions, isoform composition, and distributions). For example, PiD-like pathologies have been reported in patients with some mutations in exon 9. PSP- or CBD-like pathologies have been reported in patients with mutations that increase the splicing of exon 10 inclusions, resulting in an increased expression ratio of 4R tau isoforms. On the other hand, cases with intron 10 mutations develop a unique pathology, distinct from other sporadic 4R tauopathies (Spillantini et al., 1996; Reed et al., 1997; Bugiani et al., 1999; Spina et al., 2008; Tacik et al., 2017; Erro et al., 2019).

We have been investigating the sarkosyl-insoluble pathological tau proteins prepared from brains of patients with AD, PiD, CBD, PSP, or and FTDP-17T (intron 10 + 16), and we previously reported that the carboxyl-terminal region of tau (residues 243–406), which constitutes the trypsin-resistant core units of tau aggregates, shows different banding patterns among the diseases (Taniguchi-Watanabe et al., 2016). Furthermore, we determined the sequences and lengths of the tau regions involved in the assembly of the tau filaments in these diseases (Taniguchi-Watanabe et al., 2016). Recent cryo-EM structural analyses of tau filaments isolated from AD, PiD, and CBD brains revealed that the structures or folding of tau proteins in the filaments differs among the diseases. These findings support the idea that the pathogenesis and progression of these tauopathies are closely associated with the formation of disease-specific tau strains or folded conformers, and with cell-to-cell transmission of the pathological proteins (Goedert et al., 2018; Fitzpatrick and Saibil, 2019; Hasegawa, 2019). Furthermore, it has been pointed out that post-translational modifications (PTMs) may also play a role. Therefore, in this study, we have analyzed and compared the PTMs in the pathological tau proteins prepared from a wide range of tauopathies (AD, PSP, CBD, GGT, PiD, and FTDP-17T with MAPT intron 10 mutation) in order to establish whether the PTMs show disease-specificity.

## Materials and Methods

### Brain Tissues, Preparation of Insoluble Tau, and Immunohistochemistry

The cases selected for this study [three AD, three FTDP-17T (intron 10 + 16), three PSP, three CBD, three GGT, and three PiD cases] are listed in Table 1.

**TABLE 1:** Description of the cases used PTMs analysis.

Case No.	Age at death	Gender	Brain weight	pmi	Brain region	Neuropathological diagnosis
AD1	85	M	1146	8 h	Parietal	AD
AD2	94	F	983	11 h	Parietal	AD
AD3	61	M	nd	nd	Frontal	AD
FTDP-17T1	55	M	1240	nd	Frontal	MAPT +16
FTDP-17T2	53	M	1240	nd	Frontal	MAPT +16
FTDP-17T3	65	F	1040	nd	Frontal	MAPT +16
CBD1	73	M	1200	3 h	Frontal	CBD
CBD2	74	F	899	nd	Frontal	CBD
CBD3	74	F	nd	nd	Frontal	CBD
GGT1	74	M	1222	10 h	Frontal	GGT
GGT2	77	F	1110	2 h	Frontal	GGT
GGT3	76	F	nd	nd	Frontal	GGT
PSP1	63	M	1435	2 h	Frontal	PSP
PSP2	85	M	850	1 h	Frontal	PSP
PSP3	82	M	1280	nd	Frontal	PSP
PiD1	62	F	928	nd	Frontal	Pick
PiD2	56	M	1150	nd	Frontal	Pick
PiD3	nd	nd	nd	nd	Frontal	Pick

Sarkosyl-insoluble tau was prepared as previously described (Zhang et al., 2020); this method was developed and afforded highly purified pathological tau proteins. Briefly, 0.5 g tissues were was homogenized in 20 volumes (v/w) of extraction buffer, brought to 2% sarkosyl, and incubated for 30 min. The supernatant after a 10 min spin at 20,000 *g*, was centrifuged at 168,000 × *g* for 20 min. The pellet was resuspended in a small amount of extraction buffer and centrifuged at 9,500 *g* for 10 min. The supernatant was diluted threefold in 50 mM Tris–HCl, pH 7.5, containing 0.15 M NaCl, 10% sucrose and 0.2% sarkosyl, and spun at 168,000 *g* for 20 min.

For immunoblotting, the sarkosyl-insoluble pellet was resuspended in SDS sample buffer and the aliquots were subjected to SDS-PAGE on 4∼20% polyacrylamide gradient gel. Immunoblotting was performed with anti-tau C-terminus antibody T46 as described elsewhere (Taniguchi-Watanabe et al., 2016).

For immunohistochemistry, brain tissues were fixed by in 10% buffered formalin and embedded in paraffin, then 8-micron-thick sections were prepared and immunostained with a monoclonal antibody AT8 (Invitrogen).

### LC-MS/MS Analysis of Sarkosyl-Insoluble Tau

Sarkosyl-insoluble fractions containing 500∼5000 ng of tau were treated with 70% formic acid for 1 h at room temp, then diluted in water and dried up. For trypsin digestion, 50 mM triethylammonium bicarbonate and 1 μg of Trypsin/Lys-C Mix (Promega) were added. Each mixture was incubated at 37°C for 20 h. After tryptic digestion, 2 μL of 100 mM DTT was added to the mixture, and incubation was continued at 100°C for 5 min. Then the sample was dried and stored at −80°C until assay.

Each sample was resuspended in 0.1% formic acid and introduced into a nano-flow HPLC system, EASY-nLC 1200 (Thermo Fisher Scientific Inc., Waltham, MA, United States). A packed nano-capillary column NTCC-360/75-3-123 (0.075 mm I.D. × 125 mm L, particle diameter 3 μm, Nikkyo Technos Co., Ltd., Tokyo, Japan) was used at a flow rate of 300 nl/min with a 2–80% linear gradient of acetonitrile for 80 min. Eluted peptides were directly detected with an ion trap mass spectrometer, Q-Exactive HF (Thermo Fisher Scientific Inc., Waltham, MA, United States). For ionization, a spray voltage of 2.0 kV and a capillary temperature of 250°C was were used. The mass acquisition method consisted of one full MS survey scan with an Orbitrap resolution of 60,000, followed by an MS/MS scan of the most abundant precursor ions from the survey scan with an Orbitrap resolution of 15,000. Dynamic exclusion for the MS/MS was set to 30 s. The MS scan range of 350–1800 m/z was employed in the positive ion mode, followed by data-dependent MS/MS using the HCD operating mode on for the top 15 ions in order of abundance. The data were analyzed with Proteome Discoverer (Thermo Fisher Scientific Inc., Waltham, MA, United States), Mascot software (Matrix Science Inc., Boston, MA, United States) and Scaffold software (Proteome Software, Inc., Oregon, OR, United States). Swissprot and GenBank databases were used. Mass spectrometry data are obtained from jPOST (Japan ProteOme STandard Repository), which is certificated member of ProteomeXchange Consortium. ID number is PXD020371.

## Results and Discussion

### PTMs on Tau From Tauopathy Brains

**FIGURE 1:**
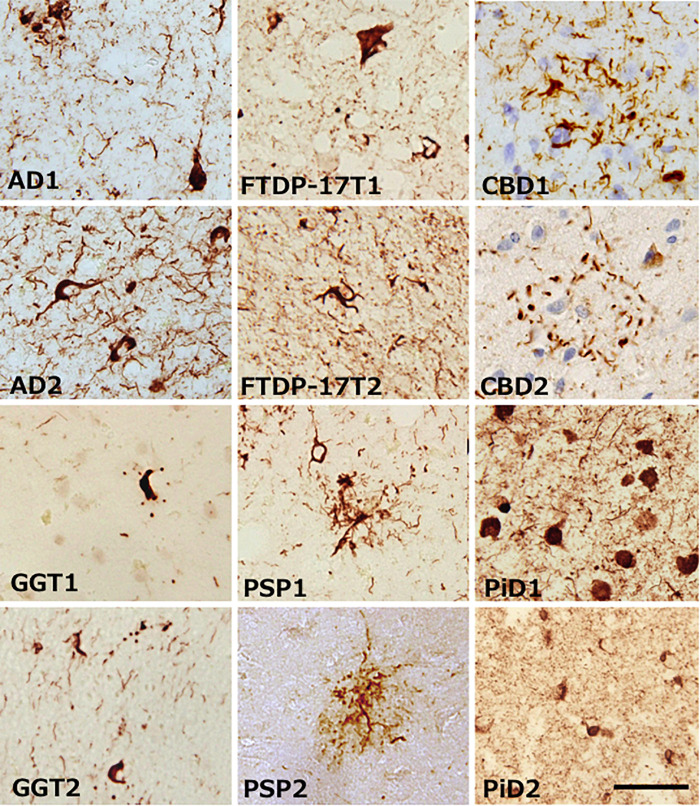
Representative images of brain tissue from patients with various tauopathies, stained with AT8 antibody. Characteristic tau inclusions of each tauopathy were detected. Black bar is indicates 50 μm.

**FIGURE 2:**
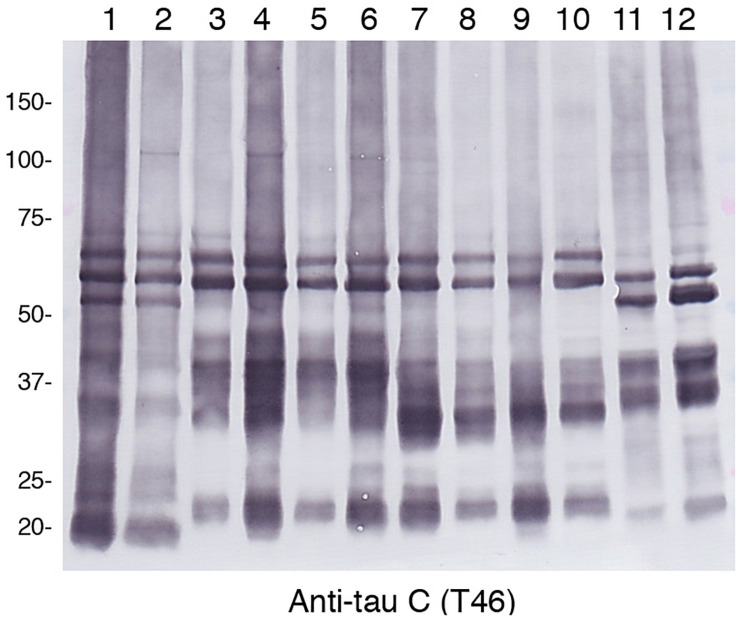
Immunoblot analysis of sarcosyl-insoluble fractions obtained from brain tissues of patients with various tauopathies. After SDS-PAGE using on 4∼20% polyacrylamide gradient gels, immunoblotting were was performed with anti-tau C-terminus antibody T46. Lane 1, AD1; lane 2, AD2; lane 3, FTDP17T1; lane 4, FTDP17T2; lane 5, CBD1; lane 6, CBD2; lane 7, GGT1; lane 8, GGT2; lane 9, PSP1; lane 10, PSP2; lane 11, PiD1; lane 12, PiD2.

Alzheimer’s disease, FTDP-17T, CBD, GGT, PSP, and PiD were neuropathologically diagnosed and biochemically confirmed by immunoblotting with anti-tau antibodies (in addition, *MAPT* intron 10 + 16 mutation was identified in the two FTDP-17T cases from Manchester Brain Bank). Two cases of each disease with abundant tau accumulation were selected for this analysis. Representative image of AT8 immunostaining of these cases are shown in Figure 1. The characteristic tau inclusions were detected in these diseases (for example, neurofibrillary tangles in AD, coiled bodies and diffuse tangles in FTDP-17T, tufted astrocytes in PSP, astrocytic plaques in CBD, globular glial inclusions in GGT, and Pick bodies in PiD) (Figure 1). Pathological tau proteins in the sarkosyl-insoluble fractions prepared from these cases were analyzed by SDS-PAGE and immunoblotting with anti-tau C-terminus antibody (T46). As shown in Figure 2 and Supplementary Figure 1, characteristic hyper-phosphorylated full-length triplet tau bands at 60, 64, and 68 kDa were observed in AD (Hasegawa, 2016, 2019). In contrast, only the upper doublet tau bands at 64 and 68 kDa were detected in the 4R tauopathies FTDP-17T (+16), CBD, GGT, and PSP, while only the lower tau doublet bands at 60 and 64 kDa were detected in PiD (Hasegawa, 2016, 2019). In addition to these full-length tau bands, many C-terminal tau fragments (CTFs) and smears were detected in all these tauopathies (Figure 2 and Supplementary Figure 1). The characteristic bands of CBD and PSP CFTs at 37 kDa CTFs and ∼33 kDa CTFs (Arai et al., 2004) were observed in both of the CBD case and both of the PSP cases in this study, respectively. The two FTDP-17T (+ 16) cases also showed a CBD-tau banding pattern, while the two GGT cases showed a PSP-tau banding pattern (Figure 2 and Supplementary Figure 1). These results are consistent with previous observations, and confirmed the strong link between neuropathological diagnosis and biochemical characterization. After denaturation of the sarkosyl-insoluble tau by formic acid treatment, the pathological tau were was digested with trypsin and the tryptic peptides were analyzed by LC-MS/MS. Identified tau peptides (FDR: 0.38%) with or without PTMs are listed in the Supplementary Table 1. The recovery of the identified tau-derived peptides in each case was 70–90% and was summarized in Supplementary Table 2.

### Common PTMs Localized in the N-terminal, Middle- and C-terminal Regions

The PTMs in each case were summarized in Table 2 (partial excerpt) and Supplementary Table 3 (all details). In these table, the total number of peptides containing each amino acid residue and the number of peptides modified with the residues are shown, and the ratios thereof (frequency of modification) are shown in the color-coding defined at the bottom of the table. The gray area in PiD is a region of amino acid residues that does not exist.

**TABLE 2:**
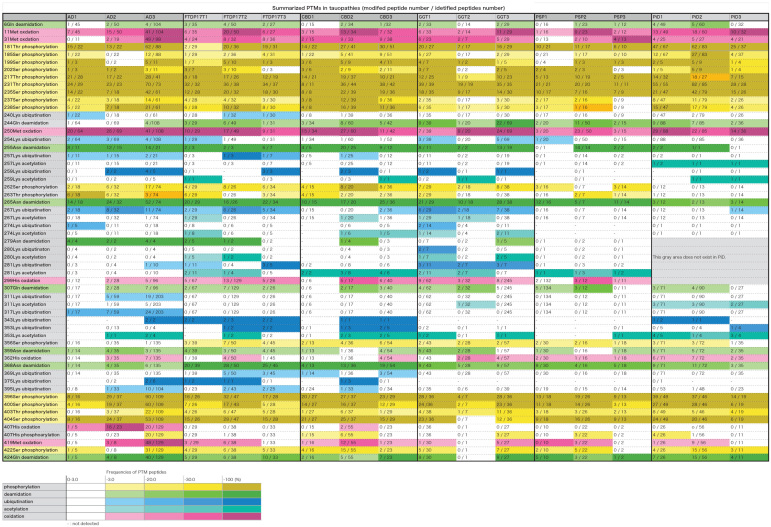
Summarized PTMs in tauopathies. In this table, the total number of peptides containing each amino acid residue and the number of peptides modified with the residues are shown, and the ratios thereof (frequency of modification) are shown in the color-coding defined at the bottom of the table. The gray area in PiD is a region of amino acid residues that does not exist.

As shown in Table 2 and Supplementary Table 3, 170 PTMs in total were identified in the sarkosyl-insoluble tau prepared from these tauopathies. Overall, the phosphorylation sites were focused in the 181–238 and 396–422 regions of the tau, corresponding N- and C-terminal flanking regions of the microtubule binding repeats. In contrast, ubiquitination sites seemed to be focused in the 254–281 and 369–395. In each tauopathy, when PTMs two or more cases are picked up, those were deamination at 6Gln, 255Asn, 265Asn, 359Asn, 368Asn, and 424Gln, oxidation at 11Met, 31Met, 250Met, 299His, and 419Met, and phosphorylation at 181Thr, 185Ser, 199pSer, 202Ser, 212Thr, 217Thr, 231Thr, 235Ser, 237Ser, 238Ser, 396Ser, 400Ser, 403Thr, 404Ser, and 422Ser were listed. These PTMs were localized in the N-terminal region, middle region (181–238) and the C-terminal region. As for Met, Asn, and Gln, oxidation and deamidation can rarely occur during experimental manipulation. Therefore, it cannot be denied that artificial modification may be included in these amino acid residues.

We previously reported that the carboxyl-terminal region (residues 243–406) of tau including the MT-binding repeat regions form the protease-resistant core units of the tau filaments, and we identified the N-terminal sequences and the trypsin-resistant regions of tau (Taniguchi-Watanabe et al., 2016). We further suggested that the assembly of these specific tau regions might be involved in the pathogenesis and progression of these distinct disease phenotypes (Taniguchi-Watanabe et al., 2016). Recent cryo-EM analyses have revealed that the R3-R4 repeat and 12 residues after the R4 repeat region in AD, the R2-R4 repeat and 12 residues after the R4 repeat region in CBD, and the R1, R3–R4 repeat and 12 residues after R4 repeat region in PiD form the cores of the tau filaments (Fitzpatrick et al., 2017; Falcon et al., 2018; Fitzpatrick and Saibil, 2019; Zhang et al., 2020), in accordance with our previous reports (Taniguchi-Watanabe et al., 2016; Hasegawa, 2019). Overall, the results indicate that most of the common PTMs detected in this study exist outside the core structures of the tau filaments, suggesting that these regions may not be involved in the strain-dependent tau filament formation.

### Characteristic Ubiquitination Sites on Tau in Each Disease

Ubiquitination occurs mainly in the region of 254–281. Furthermore, characteristic ubiquitination of disease was observed in the fibril core region. So far, the core regions revealed by cryoEM are 304–380 in AD, 274–380 in CBD, and 254–378 in PiD, which correspond to the portion between the broken lines in Supplementary Table 3 (all details). We identified ubiquitination sites characteristic of each disease in the regions forming the fibril cores, as shown in Table 2 and Supplementary Table 3. These included ubiquitination at 311Lys and 317Lys in the R3 region in AD, and ubiquitination at 343Lys, 353Lys, 369Lys, and 375Lys in the R4 and 12 residues after the R4 region in CBD. These ubiquitinated Lys corresponded to the dense part outside the tau fibril core in the data obtained by cryoEM, and it was considered that polyubiquitin chains exist in this part (Arakhamia et al., 2020; Zhang et al., 2020). Similar results have been reported elsewhere (Arakhamia et al., 2020). Similarly, in FTDP-17T, ubiquitination at 343Lys, 353Lys, 369Lys, and 370Lys in the R4 and 12 residues after the R4 region were observed. This pattern to be similar to that in CBD-tau, suggesting that the folding of tau in FTDP-17T (+16) cases seem to be similar to that in CBD. Ubiquitination at 267Lys, 274Lys, 280Lys, and 281 Lys in the R1–R2 region is characteristic of tau prepared from GGT. In PSP cases, no ubiquitination was found in the R2–4 and 12 residues after R4 region. In PiD cases, ubiquitination at 343Lys and 353Lys were found.

Based on the idea that the R2–R4 and 12 residues after R4 region are closely involved in the tau filament core formation, it seems likely that tau in GGT and PSP may have a distinct core structure different from that in AD, CBD, FTDP-17T, and PiD. It also seems likely that PTMs such as ubiquitination of tau protofilaments would affect the assembly of these protofilaments themselves (Arakhamia et al., 2020).

### Other Characteristic PTMs

Phosphorylation at 262Ser residue has been reported in various tauopathies but it was not detected in PiD using a specific monoclonal antibody (Arai et al., 2004). We have confirmed this by means of MS/MS analyses of tau prepared from various tauopathies. Furthermore, we have shown in this study phosphorylation at 356Ser residue was detected in various tauopathies, but not in AD. According to cryoEM analysis, 262Ser residues in PiD and 356Ser residue in AD are located in the center of the bent structure and are difficult to access from outside. Therefore, these PTMs are likely to have occurred after fibril formation and it is considered that phosphorylation is unlikely to occur in PiD and AD, respectively. If an antibody specific for phosphorylated Ser356 residue could be raised, it might be useful tool for distinguishing the AD-tau pathology from the other tauopathies.

Extensive deamidation of Asn279 has been detected with specific antibodies in AD, but not in other 4R tauopathies (Dan et al., 2013). This finding was also partially confirmed in this study. 279Asn was deamidated in all peptides including 279Asn in three cases of AD. Deamidation of 279Asn was detected in two cases of FTDP-17T, one case of CBD, and one case of GGT, but the ratio of the deamidated peptide was 45, 25, and 20%, which is quite different from the case of AD. In AD-tau, Asn279 is located in the outside the core of tau filaments, but it is inside the core in CBD (Fitzpatrick et al., 2017; Arakhamia et al., 2020; Zhang et al., 2020). It may reflect some structural difference in the pathological tau, causing Asn279 to be exposed away from the core region.

Phosphorylation of 217Thr has recently been reported to be a biomarker of AD (Barthelemy et al., 2020; Janelidze et al., 2020; Palmqvist et al., 2020). The average appearance rates of phosphorylated peptides at 217Threonine residue were AD, 73%; FTDP-17T, 58%; CBD, 55%; GGT, 34%; PSP, 44%; PiD, 53% (Table 2 and Supplementary Table 3). AD is clearly high and can be distinguished from other tauopathy. This is consistent with the recent report. No clear distinction could be made regarding the phosphorylation of 181T (Table 2 and Supplementary Table 3).

Furthermore, the average appearance rates of phosphorylation of 231Thr were 80–100% in all tauopathy (Table 2 and Supplementary Table 3). This means that almost all 231Thr residues are phosphorylated. Recently, phosphorylated tau profiles in soluble brain fractions in AD have been reported (Dujardin et al., 2020; Horie et al., 2020). In one report, Thr111, Ser113, Thr153, Thr181, Ser199, Ser202, Thr205, The217, Thr231, Ser262, and Ser396, and in the other, Thr181, Ser198, Ser199, Ser202, Thr217, Thr231, Ser262, Ser400, Thr403, Ser404 were phosphorylated, but in our analysis, all of these sites except Thr153 and Ser205 were phosphorylated (Table 2 and Supplementary Table 3). Furthermore, in insoluble brain fractions in AD, phosphorylation occupancies of the highest hyperphosphorylation rates (Thr181, Ser202, Thr217, and Thr231) were 28, 51, 31, and 82%, respectively (Horie et al., 2020). In our analysis, phosphorylation is progressing at these sites, and the frequencies of the phosphorylated peptides were 66% at Thr181, 37% at Ser202, 74% at Thr217, and 93% at Thr231, and the results were almost the same high value. This tendency is also seen in other tauopathy and is not considered to be a characteristic of AD alone.

### Methylation and Acetylation at Lys Residues

Lysine methylation is present at multiple sites in soluble tau isolated from cognitively normal elderly cases at locations that only partially overlapped with the distributions reported for cognitively normal middle- aged and AD cohorts, suggesting that lysine methylation may be a physiological PTM of tau protein that changes qualitatively with aging and disease (Huseby et al., 2019). Lys methylations were detected at a very small number of Lys residues in contrast to previous findings. In comparison, acetylation at Lys residues was detected in the area forming the tau fibril core (Table 2 and Supplementary Table 3). In the cases of PiD, 257Lys, 259Lys, 311Lys, and 353Lys residues were characteristically acetylated. In diseases other than PSP and PiD, the ubiquitination sites also overlap in the area forming the tau fibril core (Table 2 and Supplementary Table 3). Although ubiquitination is inhibited when acetylation occurs at Lys residue, it is unclear whether there is a regulatory relationship between the two.

### Newly Identified PTMs in Tau

In this analysis, we found some new PTMs on tau in various tauopathies. Oxidation at 299His and 362His was detected in all the diseases examined. Oxidation at 329His and 330His was detected in FTDP-17T, CBD, and PiD, while oxidation at 407His was detected in AD and CBD. Furthermore, phosphorylation at 407His was detected in all the tauopathies. MS/MS spectra of the identified His phosphorylation peptide was shown as Supplementary Figure 2. It has been reported that phosphorylation of histidine residue is rare in signal transduction systems (Fuhs and Hunter, 2017). In this case, it is located in a region where many Ser/Thr sites are heavily phosphorylated, so it is possible that the 407His is simply phosphorylated concomitantly with Ser/Thr residues, and may not have a physiological role.

### Relationship Between PTMs and Tau Filament Structures

Post-translational modifications in tau fibrils may be involved in the stability of the fibrils. PSP tau fibrils are more susceptible to enzymatic digestion than other tau fibrils in other tauopathies (Taniguchi-Watanabe et al., 2016). This is because tau fibrils of PSP have fewer PTMs than other tauopathies (Table 2 and Supplementary Table 3), which may allow digestive enzymes can to directly access the fibril core.

The use of high-performance mass spectrometers is likely to enable the detection of more minor PTMs, but it is questionable whether they are all pathophysiologically important. It may be more useful to identify PTMs common to all diseases, or disease-specific PTMs. As described above, such PTMs are likely to be closely related to the characteristic tau fibril structure in each disease. It is also likely that PTMs such as ubiquitination of tau protofilaments affect the assembly of these protofilaments themselves. Nevertheless, it is reasonable to consider that most of the PTMs occur after assembly or protofilament formation of tau, because it is unlikely that specific lysine residues on unfolded tau would be ubiquitinated by different ligases in the different diseases. It remains unclear whether PTMs affect the filament formation and contribute to form the unique structures, or whether the PTMs occur after the unique structures have been formed. Further analyses are needed to clarify resolve this question. In either case, it can be considered that the differences in PTMs reflects the differences in filament core structures.

## Data Availability Statement

The datasets presented in this study can be found in online repositories. The names of the repository/repositories and accession number(s) can be found in the article/Supplementary Material.

## Ethics Statement

The studies involving human participants were reviewed and approved by the research ethics committee of Tokyo Metropolitan Institute of Medical Science. The patients/participants provided their written informed consent to participate in this study.

## Author Contributions

MY, TM, SM, YS, IK, MO, HT, AK, AR, DM, and HM identified patients and performed the neuropathology. FK and MH carried out the biochemical analysis and the mass spectrometry analysis. All authors contributed to writing the manuscript.

## Conflict of Interest

The authors declare that the research was conducted in the absence of any commercial or financial relationships that could be construed as a potential conflict of interest.
