# Supplementary figures and images for: Comparison of Common and Disease-Specific Post-translational Modifications of Pathological Tau Associated With a Wide Range of Tauopathies
Source: Front Neurosci. 2020 Nov 4;14:581936. doi: 10.3389/fnins.2020.581936 (PMC7672045; doi:10.3389/fnins.2020.581936)

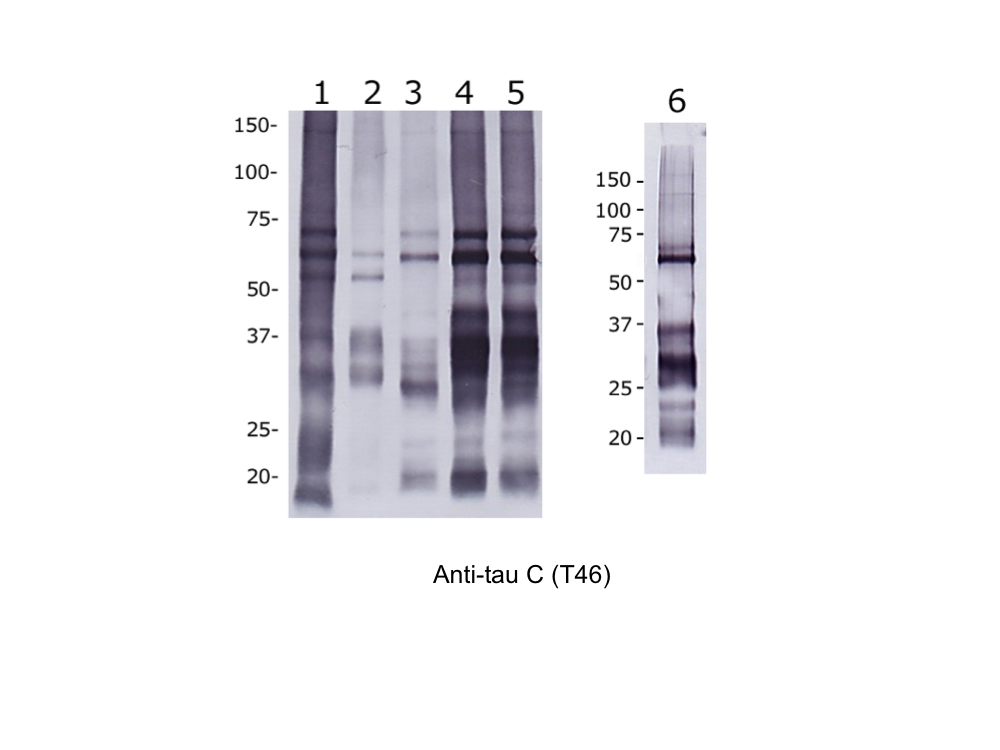

Supplement: Supplementary file 1 [file Image_1.tif]
