# Supplementary material for: Comparison of Common and Disease-Specific Post-translational Modifications of Pathological Tau Associated With a Wide Range of Tauopathies
Source: Front Neurosci. 2020 Nov 4;14:581936. doi: 10.3389/fnins.2020.581936 (PMC7672045; doi:10.3389/fnins.2020.581936)

Monoisotopic mass of neutral peptide Mr(calc): 3402.5303
Variable modifications:
H1 : Phospho (H)
S10 : Phospho (ST), with neutral losses 97.9769(shown in table), 0.0000
Ions Score: 115 **Expect:** 5.3e-011 (help)

#	a	a++	a*	a*++	b	b++	b*	b*++	Seq.	y	y++	y*	y*++	#
1	190.0376	95.5224			218.0325	109.5199			H					32
2	303.1217	152.0645			**331.1166**	166.0619			L	3088.5355	1544.7714	3071.5089	1536.2581	31
3	390.1537	195.5805			**418.1486**	209.5779			S	2975.4514	1488.2293	2958.4248	1479.7161	30
4	504.1966	252.6019	487.1701	244.0887	532.1915	266.5994	515.1650	258.0861	N	2888.4194	1444.7133	2871.3928	1436.2000	29
5	**603.2650**	**302.1362**	586.2385	293.6229	**631.2600**	316.1336	614.2334	307.6203	V	2774.3764	1387.6919	2757.3499	1379.1786	28
6	690.2971	**345.6522**	673.2705	337.1389	**718.2920**	**359.6496**	701.2654	351.1364	S	2675.3080	1338.1576	2658.2815	1329.6444	27
7	777.3291	389.1682	760.3025	380.6549	**805.3240**	403.1656	788.2975	394.6524	S	2588.2760	1294.6416	2571.2494	1286.1284	26
8	**878.3768**	439.6920	861.3502	431.1787	906.3717	453.6895	889.3451	445.1762	T	2501.2440	1251.1256	2484.2174	**1242.6123**	25
9	935.3982	468.2028	918.3717	459.6895	963.3932	482.2002	946.3666	473.6869	G	2400.1963	1200.6018	2383.1697	1192.0885	24
10	1004.4197	502.7135	987.3931	**494.2002**	**1032.4146**	516.7109	1015.3881	508.1977	S	2343.1748	1172.0910	2326.1483	**1163.5778**	23
11	1117.5038	559.2555	1100.4772	550.7422	**1145.4987**	573.2530	**1128.4721**	564.7397	I	2274.1534	1137.5803	2257.1268	1129.0670	22
12	1232.5307	616.7690	1215.5042	608.2557	**1260.5256**	**630.7664**	1243.4991	622.2532	D	2161.0693	1081.0383	2144.0427	1072.5250	21
13	1363.5712	**682.2892**	1346.5446	673.7760	**1391.5661**	696.2867	**1374.5396**	687.7734	M	2046.0423	1023.5248	2029.0158	1015.0115	20
14	1462.6396	731.8234	1445.6131	723.3102	**1490.6345**	745.8209	**1473.6080**	737.3076	V	1915.0019	**958.0046**	1897.9753	949.4913	19
15	**1577.6665**	789.3369	1560.6400	780.8236	**1605.6615**	803.3344	**1588.6349**	794.8211	D	**1815.9335**	908.4704	**1798.9069**	899.9571	18
16	1664.6986	832.8529	1647.6720	824.3397	**1692.6935**	846.8504	**1675.6669**	838.3371	S	1700.9065	850.9569	1683.8800	842.4436	17
17	1761.7513	881.3793	1744.7248	872.8660	1789.7463	895.3768	1772.7197	**886.8635**	P	**1613.8745**	**807.4409**	1596.8479	**798.9276**	16
18	1889.8099	945.4086	1872.7834	936.8953	1917.8048	959.4061	1900.7783	950.8928	Q	1516.8217	758.9145	**1499.7952**	750.4012	15
19	2002.8940	1001.9506	1985.8674	**993.4374**	2030.8889	**1015.9481**	2013.8623	1007.4348	L	**1388.7631**	694.8852	1371.7366	686.3719	14
20	2073.9311	1037.4692	2056.9045	1028.9559	2101.9260	1051.4666	2084.8995	1042.9534	A	**1275.6791**	638.3432	**1258.6525**	629.8299	13
21	2174.9788	1087.9930	2157.9522	1079.4798	2202.9737	1101.9905	2185.9471	1093.4772	T	**1204.6420**	**602.8246**	1187.6154	**594.3113**	12
22	2288.0628	1144.5351	2271.0363	1136.0218	2316.0578	1158.5325	2299.0312	1150.0192	L	**1103.5943**	**552.3008**	1086.5677	543.7875	11
23	2359.1000	1180.0536	2342.0734	1171.5403	2387.0949	1194.0511	2370.0683	1185.5378	A	**990.5102**	**495.7587**	973.4837	487.2455	10
24	2474.1269	1237.5671	2457.1003	1229.0538	2502.1218	1251.5645	2485.0953	1243.0513	D	**919.4731**	460.2402	**902.4466**	451.7269	9
25	2603.1695	1302.0884	2586.1429	**1293.5751**	2631.1644	1316.0858	2614.1379	1307.5726	E	**804.4462**	402.7267	**787.4196**	394.2134	8
26	2702.2379	1351.6226	2685.2114	1343.1093	2730.2328	1365.6200	2713.2063	1357.1068	V	**675.4036**	338.2054	658.3770	329.6921	7
27	2789.2699	1395.1386	2772.2434	1386.6253	2817.2648	1409.1361	2800.2383	1400.6228	S	**576.3352**	288.6712	559.3086	280.1579	6
28	2860.3070	1430.6572	2843.2805	1422.1439	2888.3020	1444.6546	2871.2754	1436.1413	A	**489.3031**	245.1552	472.2766	236.6419	5
29	2947.3391	1474.1732	2930.3125	1465.6599	2975.3340	1488.1706	2958.3074	1479.6574	S	**418.2660**	209.6366	401.2395	201.1234	4
30	3060.4231	1530.7152	3043.3966	1522.2019	3088.4181	1544.7127	3071.3915	1536.1994	L	**331.2340**	166.1206	314.2074	157.6074	3
31	3131.4603	1566.2338	3114.4337	1557.7205	3159.4552	1580.2312	3142.4286	1571.7179	A	218.1499	109.5786	201.1234	101.0653	2
32									K	147.1128	74.0600	130.0863	65.5468	1

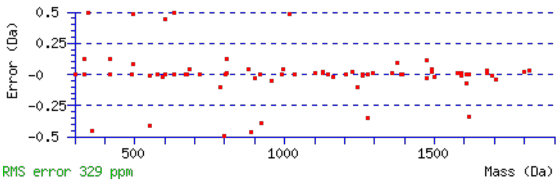

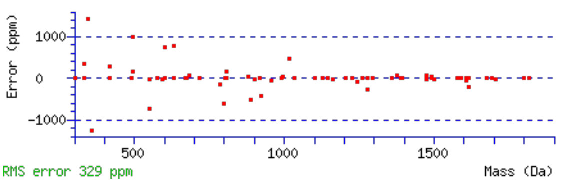

Supplement: Supplementary file 2 [file Image_2.pdf]
