# Supplementary material for: Comparison of Common and Disease-Specific Post-translational Modifications of Pathological Tau Associated With a Wide Range of Tauopathies
Source: Front Neurosci. 2020 Nov 4;14:581936. doi: 10.3389/fnins.2020.581936 (PMC7672045; doi:10.3389/fnins.2020.581936)
Supplement: Supplementary file 6 [file Data_Sheet_1.docx]

**Supplemental Table 1**

Identified peptides list with or without PTMs. The parameters of the mascot analysis are shown below.

Type of search : MS/MS Ion Search

Enzyme : Trypsin

Variable modifications : Acetyl (K), Oxidation (HW), Deamidated (NQ), Gln->pyro-Glu (N-term Q), Methyl (K), GlyGly (K), Oxidation (M), Phospho (ST), Phospho (H)

Mass values : Monoisotopic

Protein mass : Unrestricted

Peptide mass tolerance : ± 10 ppm

Fragment mass tolerance : ± 0.5 Da

Max missed cleavages : 3

**Supplemental Table 2**

Summary of tau sequence coverages.

**Supplemental Table 3**

Summarized PTMs in tauopathies. In this table, the total number of peptides containing each amino acid residue and the number of peptides modified with the residues are shown, and the ratios thereof (frequency of modification) are shown in the color-coding defined at the bottom of the table. The gray area in PiD is a region of amino acid residues that does not exist.

**Supplemental Figure 1**

Immunoblot analysis of sarcosyl-insoluble fractions obtained from brain tissues of patients with various tauopathies. After SDS-PAGE using on 4~20% polyacrylamide gradient gels, immunoblotting were was performed with anti-tau C-terminus antibody T46 as described [24]. Lane 1: AD3; lane 2: PiD3; lane 3: PSP3; lane 4: CBD3; lane 5: FTDP-17T3; lane 6: GGT3.

**Supplemental Figure 2**

Identified MS/MS spectrums derived from HLSNVSSTGSIDMVDSPQLATLADEVSASLAK peptide. Red number is the identified MS/MS spectra of each fragment.

331.1166 was identified as the spectrum of the phosphorylated His-Leu-Ser fragment, and 418.1486 was identified as the spectrum of the phosphorylated His-Leu-Ser fragment. Therefore, it is unlikely that the phosphorylation of Ser is mistakenly recognized as the phosphorylation of His in this portion. This means that His of this peptide is definitely phosphorylated.
